# Comparative efficacy and tolerability of currently approved incretin mimetics: A systematic analysis of placebo‐controlled clinical trials

**DOI:** 10.1111/dom.16398

**Published:** 2025-04-11

**Authors:** Yu Mi Kang, Viktoria Punov, Soo Lim, Michael A. Nauck

**Affiliations:** ^1^ Division of Endocrinology, Diabetes and Hypertension and TIMI Study Group Brigham and Women's Hospital, Harvard Medical School Boston Massachusetts USA; ^2^ Diabetes, Endocrinology, Metabolism Section Medical Department I, Josef‐Hospital Ruhr University Bochum Bochum Germany; ^3^ Seoul National University College of Medicine Seoul National University Bundang Hospital Seongnam South Korea; ^4^ Institute for Clinical Chemistry and Laboratory Medicine University Medicine Greifswald Greifswald Germany

**Keywords:** GLP‐1 analogue, incretin therapy, systematic review, type 2 diabetes, weight management

## Abstract

**Aims:**

This study compares the therapeutic efficacy, gastrointestinal (GI) adverse event (AE) rates and the relationship between the therapeutic efficacy and GI AEs in randomized, placebo‐controlled clinical trials (RCTs) of GLP‐1 RAs and the dual GLP‐1/GIP agonist tirzepatide.

**Materials and Methods:**

A systematic PubMed search identified 38 phase 3 or 4 placebo‐controlled RCTs of exenatide (b.i.d. and q.w.), lixisenatide, liraglutide, dulaglutide, albiglutide, semaglutide (s.c. and oral) and tirzepatide with a total of 16 660 individuals with type 2 diabetes (T2D) across 104 study arms. Changes in HbA1c, fasting plasma glucose and body weight and the proportion of GI AEs (nausea, vomiting or diarrhoea) were calculated by agent, preparation and dose. The correlation between odds ratios (ORs) for GI AEs and the magnitude of therapeutic efficacy was assessed in a linear regression analysis.

**Results:**

Baseline characteristics were similar across studies: mean age 57 ± 10 years, diabetes duration 8 ± 6 years, body mass index (BMI) 31.9 ± 5.8 kg/m^2^ and HbA1c 8.2% ± 0.9%. HbA1c reductions ranged from −0.63% ± 0.03% (lixisenatide, 20 μg q.d.) to −1.79% ± 0.09% (tirzepatide, 15 mg q.w.; *p* < 0.0001). Weight reductions ranged from −0.75 ± 0.10 kg to −9.65 ± 0.56 kg. Despite the high variability in therapeutic efficacy, ORs for GI AEs were similar across compounds/preparations.

**Conclusions:**

The magnitude of efficacy for intended therapeutic actions (HbA1c and body weight reduction) varied widely between incretin mimetic glucose‐lowering agents. However, larger therapeutic efficacy was not systematically associated with higher GI AE or drug discontinuation rates, indicating better tolerability of the more effective agents/preparations.

## 1 INTRODUCTION

Incretin mimetics are glucose‐lowering medications widely used for the management of type 2 diabetes mellitus (T2D), originally developed based on the glucose‐ and weight‐reducing effects of glucagon‐like peptide‐1 (GLP‐1).^1^, ^2^, ^3^ The newest addition to this class is tirzepatide, the first unimolecular co‐agonist acting on both GLP‐1 and glucose‐dependent insulinotropic polypeptide (GIP) receptors.^4^, ^5^, ^6^ While the additive effect of GIP receptor agonism is still being explored, newer incretin mimetics generally show greater reductions in HbA1c and body weight compared with GLP‐1 receptor agonists (GLP‐1 RAs) developed earlier.^7^, ^8^, ^9^

Head‐to‐head comparisons^7^, ^10^, ^11^, ^12^, ^13^, ^14^, ^15^, ^16^, ^17^, ^18^, ^19^ and network meta‐analyses^20^, ^21^ have shown significant variations in the efficacy of the incretin mimetics in reducing HbA_1c_, fasting plasma glucose (FPG) and body weight reductions, which have informed their differential use based on individual patient needs. However, another important consideration that influences the selection of these agents is the risk for adverse events (AEs), particularly gastrointestinal (GI) side effects such as nausea, vomiting and diarrhoea.^22^ Potential differences in the GI AEs and their relationship with therapeutic efficacy across trials have not received the same attention and have not—so far—been systematically explored.

To address this knowledge gap, the current study compares therapeutic efficacy (i.e., effect size for intended therapeutic actions—HbA1c and body weight reduction), the risk of GI AE versus placebo treatment and their correlation across phase 3 and 4 randomized, placebo‐controlled clinical trials studying various incretin‐based glucose‐lowering agents used for the treatment of type 2 diabetes (T2D).

## 2 METHODS

### 2.1 Search strategy and selection criteria

To investigate outcomes associated with currently approved doses of incretin mimetics, we identified primary publications of Phase 3 and 4 placebo‐controlled clinical trials of approved GLP‐1 receptor agonists from pivotal trial programmes through a PubMed search using pre‐specified terms (Supplementary Figure 1). The analysis protocol was registered with PROSPERO (CRD42023398350). A parallel search through ClinicalTrials.gov was conducted. The search terms included specific agents and their pivotal study programmes: Exenatide twice daily/AMIGO; lixisenatide/Get Goal; liraglutide/LEAD; exenatide once weekly/DURATION; dulaglutide/AWARD; albiglutide/HARMONY; semaglutide (subcutaneous injection)/SUSTAIN; oral semaglutide/PIONEER; tirzepatide/SURPASS. In addition, studies initiated by pharmaceutical companies for the aforementioned agents on a background of other glucose‐lowering medications, which were not available at the time of the original study programme (e.g., sodium‐glucose co‐transporter‐2 [SGLT‐2] inhibitor for liraglutide^23^) were included. Studies focusing on specific subpopulations (e.g., those with chronic kidney disease or steatotic liver disease) were excluded to maximize generalizability. For trials reporting multiple follow‐up periods, data were analysed up to the pre‐specified primary endpoint. This analysis was conducted in accordance with the Preferred Reporting Items for Systematic Reviews and Meta‐analyses (PRISMA) reporting guideline (Supplementary Figure 1). All eligible studies were reviewed independently by 2 authors (Y.M.K. and V.P.), and discrepancies were resolved through discussion. Risk‐of‐bias assessments were also completed by 2 authors (Y.M.K and M.A.N) using the Cochrane risk‐of‐bias assessment tool version 2 (Supplementary Figure 2).

### 2.2 Exposure

Participants were grouped by compound, preparation (subcutaneous injection vs. oral for semaglutide) and dose. Placebo arms were pooled per compound and preparation for comparison.

### 2.3 Outcomes

Primary efficacy outcomes included placebo‐corrected absolute changes in HbA1c, FPG and body weight from baseline, as well as the proportion of participants achieving predefined HbA1c targets (<7.0% or ≤6.5%). Safety outcomes included the proportion of participants reporting GI AEs such as nausea, vomiting or diarrhoea and the proportion of participants who discontinued treatment due to AEs or any reason.

### 2.4 Data analysis

Baseline characteristics and primary efficacy and safety outcomes were assessed using weighted means and pooled standard deviations (SDs). Pooled effect sizes for primary efficacy outcomes were compared across agents at their highest approved doses, with lixisenatide‐the least effective GLP‐1 RA—used as the reference. Odds ratios (ORs) for GI AEs relative to placebo were calculated for each agent/preparation/dose. Dose–response relationships were evaluated for all agents with two or more approved dosage regimens, considering both efficacy and safety outcomes. Effect sizes for efficacy outcomes were plotted against ORs for GI AEs and treatment discontinuation rates to assess the degree of potential correlations between therapeutic efficacy and AE or discontinuation rates. ORs for the most commonly reported GI AEs (nausea and vomiting) and for discontinuation of randomized treatment (for any reason and because of AEs) were selected as the primary measure of association for treatment versus placebo. For comparative efficacy analyses, only data from the highest approved doses of GLP‐1 RAs and tirzepatide were included.

Baseline characteristics and efficacy were compared using one‐way ANOVA for continuous variables, followed by post hoc pairwise comparisons to identify differences between agents. Dose–response relationships were evaluated using unpaired t‐tests comparing the respective lowest and highest doses. Categorical variables, such as rates of HbA1c target achievement and the proportion of participants reporting GI AEs, were analysed using contingency tables, with Fisher's exact test applied for 2 × 2 tables and the chi‐square test for larger tables. Post hoc pairwise comparisons for categorical variables were conducted using Fisher's exact test. Linear regression analyses were performed to assess the relationship between the magnitude of therapeutic efficacy and ORs for GI AEs. Regression results are reported with regression equations, correlation coefficients (*r* ^2^) and *p*‐values, with significance defined as *p* < 0.05. Statistical analysis and graphical presentations were performed with GraphPad Prism version 10 for Windows (www.graphpad.com). R version 4.4.3 was used to assess heterogeneity using Cochrane's *Q* test and *I* ^2^ statistics among study arms receiving the highest approved dose of each compound.

## 3 RESULTS

### 3.1 Baseline population characteristics

A total of 38 clinical trials, involving 16 660 participants allocated to 104 study arms, were included in the analysis. Details of the included studies are listed in Supplementary Table 1. Baseline characteristics of all participants, both overall and grouped by the type of incretin mimetic studied, are presented in Table 1. Across all participants, 47.4% were female, with a weighted mean (± pooled SD) age of 56.5 ± 9.8 years, diabetes duration of 8.4 ± 5.8 years, body mass index of 31.9 ± 5.8 kg/m^2^, body weight of 89.3 ± 19.2 kg, HbA1c of 8.2% ± 0.6% and FPG of 9.3 ± 1.7 mmol/L (Table 1). While the large sample sizes in each trial led to statistically significant differences in baseline characteristics such as HbA1c, FPG and body weight, the baseline characteristics were sufficiently similar (Table 1) to allow a meaningful comparison of therapeutic efficacy (change vs. baseline, placebo‐subtracted).

**TABLE 1:** Pooled participant characteristics by incretin mimetic agent/preparation in placebo‐controlled trials studying effects in subjects with type 2 diabetes.

	Exenatide b.i.d (a)	Lixisenatide (b)	Liraglutide (c)	Exenatide q.w. (d)	Dulaglutide (e)	Albiglutide (f)	Semaglutide s.c. (g)	Semaglutide oral (h)	Tirzepatide (h)	All compounds/preparations	Significance (*p*‐value)
*n*	1446	4062	2834	242	1806	1392	1085	2380	953	16 660	n.a.
% female	41.7	52.3	46.4	47.9	46.7	45.8	44.0	46.2	46.4	47.4	<0.0001
Age (years)	54.8 ± 10.1	56.0 ± 9.9^a^	56.1 ± 9.8^a^	56.4 ± 9.9^a^	57.1 ± 9.6^a,b,c^	54.4 ± 8.6^b,c,d,e^	56.4 ± 9.6^a,f^	59.3 ± 9.9^a,b,c,d,e,f,g^	57.4 ± 10.9^a,b,c,f,h^	56.5 ± 9.8	<0.0001
Diabetes duration (years)	7–5 ± 5.9	8.0 ± 5.8	7.9 ± 5.4	10.3 ± 5.4^a,b,c^	9.5 ± 6.0^a,b,c,e^	6.7 ± 4.7^a,b,c,e,f^	9.0 ± 5.8^a,b,c,e,g^	9.6 ± 6.6^a,b,c,g^	9.0 ± 6.4^a,b,c,e,g^	8.4 ± 5.8	<0.0001
BMI (kg/m^2^)	33.7 ± 5.7	31.1 ± 6.0^a^	31.3 ± 5.1^a^	32.0 ± 5.1	32.6 ± 5.5^b^	33.2 ± 5.4^b,c^	32.4 ± 6.6	31.3 ± 6.2^a,f^	32.6 ± 6.3	31.9 ± 5.8	<0.0001
Body weight (kg)	96.9 ± 19.6	84.9 ± 20.0^a^	88.0 ± 18.3^a,b^	92.3 ± 15.3^a,b,c^	92.9 ± 19.5^a,b,c^	92.1 ± 17.0^a,b,c^	91.6 ± 19.6^a,b,c^	87.0 ± 20.2^a,b,d,e,f,g^	91.3 ± 20.8^a,b,c,h^	89.3 ± 19.2	<0.0001
HbA_1c_ (%)	8.5 ± 0.7	8.2 ± 0.9^a^	8.4 ± 0.6^a,b^	8.9 ± 0.6^a,b,c^	8.2 ± 0.7^a,c,d^	8.1 ± 0.5^a,c,d^	8.2 ± 1.0^a,c,d^	8.0 ± 0.5^a,b,c,d,e,f,g^	8.1 ± 0.6^a,c,d,g^	8.2 ± 0.6	<0.0001
FPG (mmol/L)	9.9 ± 2.1	9.0 ± 2.3^a^	9.8 ± 1.7^b^	9.9 ± 1.8^b^	9.0 ± 2.1^a,c,d^	9.3 ± 1.5^a,b,c,d,e^	9.0 ± 1.9^a,c,f^	8.9 ± 1.7^a,b,c,d,e,f,g^	8.8 ± 2.1^a,b,c,d,e,f,g^	9.3 ± 1.7^d^	<0.0001
Proportion treated with (%)										
Metformin	73.9	69.7^a^	85.1^a,b^	100.0^a,b,c^	80.4^a,b,c,d^	74.0^a,b,c,e^	50.3^a,b,c,d,e,f^	39.5^a,b,c,d,e,f,g^	41.3^a,b,c,e,g,h^	69.2	<0.0001
SU/meglitinide	76.8	27.1^a^	41.2^a,b^	0.0^a,b,c^	16.6^a,b,c,d^	0.0^a,b,c,e^	3.7^a,b,c,d,e,f^	5.6^a,b,c,d,e,f,g^	0.0^a,b,c,g,h^	22.5	<0.0001
Insulin	0.0	31.8^a^	0.0	65.6^a,c^	38.8^a,c,d^	0.0^b,e^	32.5^a,c,d,e,f^	33.7^a,b,c,d,e,f^	49.8^a,c,d,e,g,h^	23.8	<0.0001
DPP‐4 inhibitor	0.0	0.0	0.0	0.0	0.0^d^	0.0	0.0^b^	2.4^a,b,c,d,e,f,g^	0.0^h^	0.3	<0.0001
SGLT‐2 inhibitor	0.0	0.0	10.7^a,b^	0.0^c^	0.0^b,c^	0.0^c^	27.7^a,b,c,d,e,f^	5.5^a,b,c,d,e,f,g^	0.0^c,g,h^	4.3	<0.0001
TZD	0.0	0.0	15.6^a,b^	0.0^c^	0.0^c^	21.6^a,b,c,d,e^	0.0^c,f^	0.0^c,f^	0.0^c,f^	4.3	<0.0001
α‐GI	0.0	0.0	0.0	0.0	0.0	0.0	0.0	0.3^a,b,c,e^	0.0	0.0	<0.0001

*Note*: Pooled weighted means ± standard deviations (continuous variables) or proportions (percentages; categorical variables); Statistical analysis: one‐way ANOVA and post hoc tests (continuous variables) or contingency table analysis (*χ* ^2^ for overall comparisons and Fisher's exact test for 2 × 2 tables, e.g., for post hoc tests). ^a–i^Significant differences (*p* < 0.05) versus agents represented by this letter (*p* < 0.05 by post hoc test).

Abbreviations: BMI, body mass index; FPG, fasting plasma glucose; GI, glucosidase inhibitors; SU, sulfonylureas; TZD, thiazolidinedione.

Differences were observed in the use of background glucose‐lowering medications across groups (Table 1). Metformin was the most commonly used medication overall, though its use was lower in studies involving newer incretin mimetics such as oral or subcutaneous semaglutide and tirzepatide. Conversely, sulfonylureas and meglitinides were more frequently used in trials involving earlier‐approved agents such as lixisenatide and liraglutide. Basal insulin use varied widely, ranging from 0% to 65.6% across studies. SGLT‐2 inhibitors were primarily used in studies involving liraglutide and both forms of semaglutide, while thiazolidinediones were used only in studies with liraglutide and albiglutide. The use of dipeptidyl peptidase‐4 (DPP‐4) inhibitors and α‐glucosidase inhibitors was rare overall.

### 3.2 Primary efficacy outcomes

#### 3.2.1 Changes in HbA1c and FPG

First, a separate analysis of studies using incretin‐based glucose‐lowering medications either on a background of basal insulin therapy or without insulin was performed (Supplementary Figure 3). Similar reductions in HbA1c and consistent trends across agents and formulations were observed regardless of the background use of basal insulin. Thus, a pooled analysis disregarding a co‐medication with basal insulin seemed appropriate.

**FIGURE 1:**
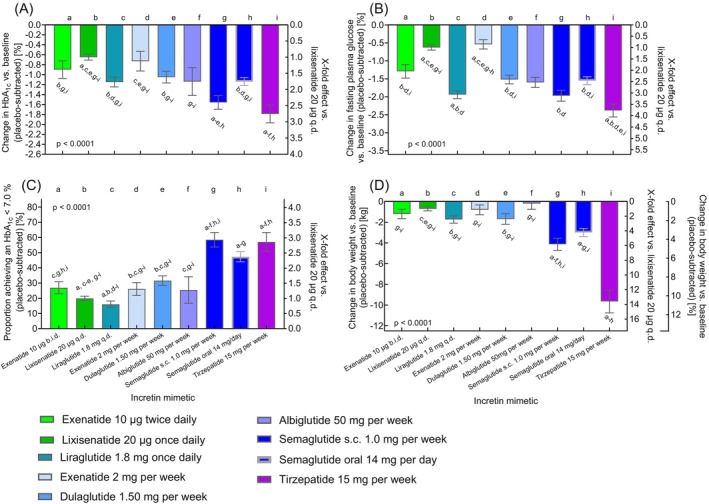
Comparative therapeutic efficacy of approved incretin mimetics in glycaemic control and body weight reduction. Pooled effect sizes from placebo‐controlled studies are shown for the highest approved doses of exenatide b.i.d., lixisenatide, liraglutide, exenatide q.w., dulaglutide, albiglutide, semaglutide (s.c. and p.o.), and tirzepatide. Placebo‐subtracted (A) HbA1c reduction, (B) fasting plasma glucose reduction, (C) proportion achieving HbA1c < 7.0% (<53 mmol/mol) and (D) body weight reduction are displayed as fold change (mean ± 95% confidence intervals) relative to lixisenatide 20 μg/day (reference treatment). Non‐overlapping 95% confidence intervals indicate statistically significant differences.

Figure 1 illustrates differences in therapeutic efficacy across injectable compounds at their highest approved doses, expressed as fold change relative to lixisenatide 20 μg q.d., the least effective compound. A general trend towards greater efficacy was observed with more recently developed compounds and formulations (Figure 1A). Compared to lixisenatide, reductions in HbA1c with liraglutide (1.8 mg/day), semaglutide s.c. (1.0 mg/week) and tirzepatide (15 mg/week) were 1.71‐, 2.3‐ and 2.62‐fold greater, respectively (*p* < 0.05 for all comparisons). Results for FPG concentrations were similar to those reported for HbA1c (Figure 1B), except for a rather prominent effect of liraglutide. HbA1c target achievement (<7.0%) mirrored HbA1c results (Figure 1C). Semaglutide s.c. was the most effective selective GLP‐1 RA, and results for oral semaglutide showed somewhat weaker effects. Overall, tirzepatide was characterized by the largest effect sizes (Figure 1) Of note, statistical heterogeneity in the pooled effect sizes of therapeutic efficacy outcomes was highly variable across study arms involving the highest approved doses of each incretin mimetic (*I* ^2^ range, 0%–97% across study arms; Supplementary Table 2).

Most agents with multiple approved doses demonstrated a dose–response relationship in HbA1c reduction when comparing the highest and lowest dose groups (Supplementary Figure 3A–D). Similar trends were observed for albiglutide, subcutaneous semaglutide and tirzepatide, although the statistical significance varied across outcomes.

#### 3.2.2 Proportion of participants achieving glycaemic targets

The improvements in glycaemic control were also reflected in the proportion of participants achieving glycaemic targets, such as an HbA1c < 7.0% (Figure 1C). The placebo‐corrected proportion of participants achieving this goal ranged from 19.1% with lixisenatide, 58.8% with semaglutide s.c. and 62.4% with tirzepatide, underscoring the higher efficacy of more recently introduced agents. The overall pattern of goal achievement across agents and doses (Supplementary Figure 4C) was consistent with the placebo‐subtracted HbA1c changes observed in (Supplementary Figure 4A). Similar trends were observed for HbA1c targets of <7.0% and ≤6.5%, as shown in Supplementary Figure 5.

#### 3.2.3 Changes in body weight

Among the four primary efficacy outcomes, the largest variability in effect size with incretin mimetics was observed for placebo‐corrected body weight reductions (Figure 1D; Supplementary Figure 3D). Reductions ranged from −0.2 kg with albiglutide to −0.7 kg with lixisenatide, −4.1 kg with semaglutide s.c. and −9.7 kg with tirzepatide. Oral semaglutide elicited less pronounced weight reductions compared with subcutaneous semaglutide. Significant dose–response relationships were observed for most agents and formulations, except for exenatide b.i.d. and albiglutide (Supplementary Figure 3D).

Relative to lixisenatide, the reductions in body weight with liraglutide (1.8 mg/day), semaglutide s.c. (1.0 mg/week) and tirzepatide (15 mg/week) were 1.71‐, 4,16‐ and 11.52‐fold greater, respectively (Figure 1D). Collectively, these findings highlight substantially more effective weight reduction achieved with more recently developed incretin mimetics.

### 3.3 Safety and tolerability outcomes

#### 3.3.1 Proportions of participants reporting GI AEs

**FIGURE 2:**
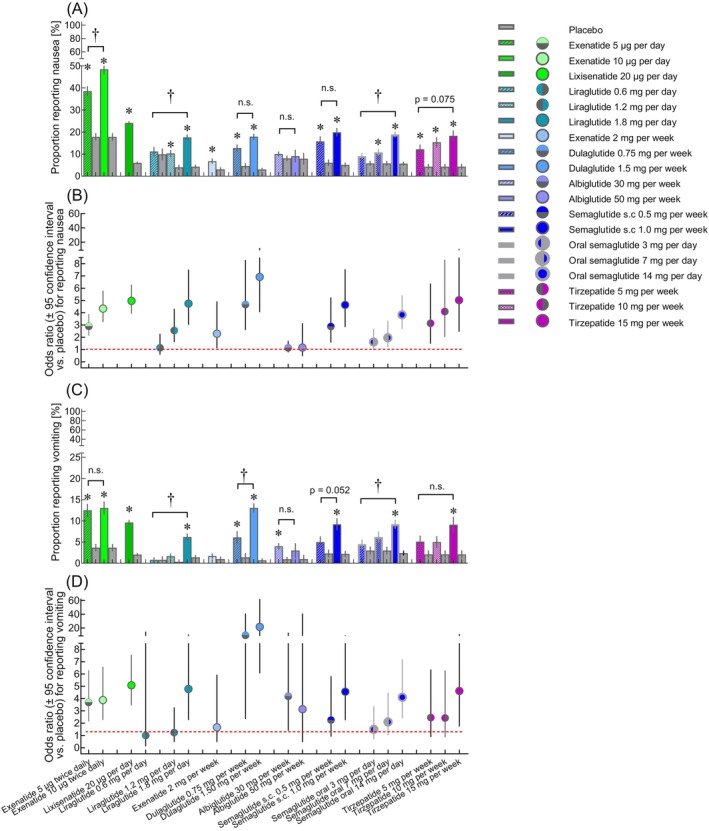
Incidence of nausea and vomiting in clinical studies of approved incretin mimetics. Pooled effect sizes from placebo‐controlled studies are shown for all approved doses of exenatide b.i.d., lixisenatide, liraglutide, exenatide q.w., dulaglutide, albiglutide, semaglutide (s.c. and p.o.) and tirzepatide. Proportions of participants reporting nausea (A) and vomiting (C) are presented for actively treated groups (colour‐coded) and placebo groups (grey). Mean values ± standard errors of the mean (SEM) are displayed. Odds ratios (ORs) with 95% confidence intervals for active versus placebo treatment are shown for nausea (B) and vomiting (D). Fisher's exact test was used for contingency table comparisons between active and placebo treatment and between the lowest and highest approved doses of each agent. Asterisks (*) indicate a significant difference (*p* < 0.05) versus placebo; daggers (†) indicate a significant dose–response relationship (*p* < 0.05) between the highest and lowest doses of the respective agent.

The proportion of participants reporting nausea was higher among those receiving incretin mimetics compared with placebo (19.3% vs. 6.5%), corresponding to an OR of 3.42 (95% CI: 3.03–3.87; Table 2). Reporting rates for nausea varied considerably by agent, ranging from 7% to 49% for exenatide once weekly, while rates ranged from 3% to 9% in the corresponding placebo group participants (Figure 2A). Nausea was reported more frequently in the active treatment arms compared with placebo across all agents except albiglutide. A statistically significant dose–response relationship was observed with exenatide b.i.d., liraglutide and oral semaglutide, whereas nonsignificant trends were noted for dulaglutide, semaglutide s.c. and tirzepatide (Figure 2).

**TABLE 2:** Adverse events (AEs; nausea, vomiting, diarrhoea, hypoglycaemia [any severity or severe]) and discontinuation of randomized treatment in pooled placebo‐controlled studies with incretin mimetics (GLP‐1 receptor agonists and the GIP/GLP‐1 receptor co‐agonist tirzepatide).

Adverse event	Treatment	Number reporting/not reporting this AE	Percentage reporting this AEs	Absolute difference between active and placebo treatment placebo (%)	Odds ratio	95% confidence interval	Significance (*p*‐value)
Nausea	Incretin mimetics	2072/8675	19.3	12.8	3.42	3.03; 3.87	<0.0001
	Placebo	327/4693	6.5				
Vomiting	Incretin mimetics	759/9170	7.6	5.6	4.07	3.28; 5.07	<0.0001
	Placebo	92/4528	2.0				
Diarrhoea	Incretin mimetics	960/9243	9.4	5.0	1.83	1.59; 2.11	<0.0001
	Placebo	2 584 542	5.4				
Discontinuation of randomized drug (any reason)	Incretin mimetics	1467/10 010	12.8	−0.7	0.94	0.87; 1.02	0.15
	Placebo	1165/7478	13.5				
Discontinuation of randomized drug (AEs)	Incretin mimetics	701/107 172	6.5	3.6	2.37	2.04; 2.75	<0.0001

Vomiting was reported less frequently than nausea among participants receiving incretin mimetics (7.6% vs. 2.0% in placebo‐treated participants), but the OR for vomiting was similar (4.07, 95% CI: 3.28–5.07; Table 2). All agents had at least one dose associated with a significantly higher prevalence of vomiting compared with placebo, but a significant dose–response relationship was only observed with liraglutide and oral semaglutide (Figure 2B).

Diarrhoea was reported in 9.4% of participants receiving incretin mimetics and 5.4% of those receiving placebo, with an OR of 1.83 (95% CI: 1.59–2.11; Table 2), relatively lower than that observed for nausea and vomiting. A significant dose–response relationship was observed only for dulaglutide (Supplementary Figure 6).

Given the variability in GI AE rates among placebo‐treated participants, ORs were calculated for each incretin mimetic compound/preparation at all approved doses (Figure 2B,D and Supplementary Figure 6B). While the 95% CIs largely overlapped, significant differences were observed, particularly between the highest doses of exenatide b.i.d., liraglutide, dulaglutide, semaglutide (s.c. and oral) and tirzepatide compared with lower doses of liraglutide, albiglutide and oral semaglutide. Overlap was more pronounced for vomiting (Figure 2B). Although there were trends towards dose–response relationships, most of them did not reach statistical significance. Notably, ORs for GI AEs in dulaglutide studies appeared higher compared with other studies (Figure 2), likely because some studies reported no nausea, vomiting or diarrhoea in the placebo group (details not shown).

#### 3.3.2 Treatment discontinuation

The overall proportion of participants discontinuing randomized treatment for any reason was similar between the active treatment and placebo groups (12.8% vs. 13.5%, OR 0.94, 95% CI: 0.87–1.02; Table 2). A dose–response relationship in the proportion of participants discontinuing treatment for any reason was observed for albiglutide, oral semaglutide and tirzepatide (Supplementary Figure 7A). However, the ORs for all‐cause treatment discontinuation varied across agents without a consistent pattern across agents (Supplementary Figure 7B). In contrast, treatment discontinuation due to adverse events (AEs) was consistently higher among participants receiving incretin mimetics compared with to placebo, with evidence of a dose–response relationship across most agents (Supplementary Figure 7C,D). The proportion discontinuing treatment due to AEs was 6.5% in the active treatment group and 2.9% in the placebo group, for an OR of 2.37 (95% CI: 2.04–2.75; Table 2).

#### 3.3.3 Relationship between the therapeutic efficacy of incretin mimetic agents and GI AEs

**FIGURE 3:**
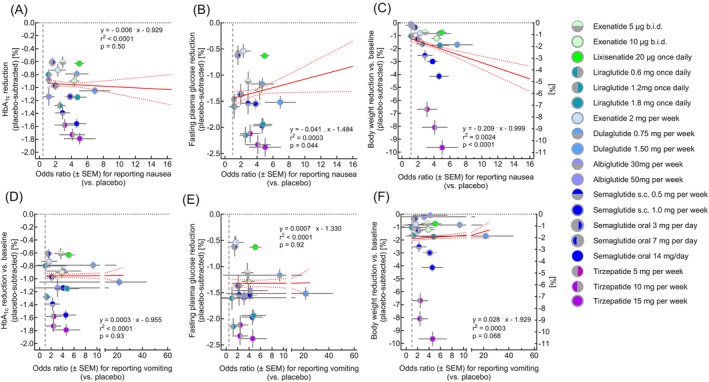
Relationship between gastrointestinal adverse events and efficacy in reducing HbA1c, fasting plasma glucose and body weight. The *x*‐axis represents odds ratios (ORs) (±SEM) for nausea (A–C) or vomiting (D–F) with active treatment relative to placebo. The *y*‐axis displays pooled effect sizes for placebo‐subtracted reductions in HbA1c, fasting plasma glucose and body weight from studies evaluating all approved doses of exenatide b.i.d., lixisenatide, liraglutide, exenatide q.w., dulaglutide, albiglutide, semaglutide (s.c. and p.o.) and tirzepatide. Linear regression results include the regression line (±95% confidence intervals, in red), regression equation, coefficient of determination (*r* ^2^) and *p*‐value.

ORs for each GI AE were plotted against each primary efficacy endpoint to assess correlations across agents and doses (Figure 3). Although some variability in ORs for GI AEs was observed, the overall range was similar across incretin mimetics, regardless of efficacy. Thus, the inspection of Figure 3 suggests that the ORs for adverse events are more or less stacked one above the other on the *x*‐axis, with some variation mainly reflecting the dose–response relationships (Figure 2), while the effect sizes are widely spread along the *y*‐axis. Linear regression analysis showed weak, but nominally significant associations between ORs for nausea and reductions in HbA1c, FPG and body weight (Figure 3A–C), as well as between ORs for vomiting and reductions in body weight (Figure 3F). However, the magnitude of these correlations was small, with *r* ^2^ values <0.016 and often <0.0001, indicating that GI AEs accounted for less than 1.6% of the variance in efficacy outcomes. Notably, data points representing highly efficacious incretin mimetics, such as s.c. and p.o. semaglutide and tirzepatide, consistently plotted farthest from the regression lines towards the lower‐left quadrant in Figure 3.

Similar analyses for diarrhoea and treatment discontinuation, presented in Supplementary Figures 8 and 9, support the conclusions derived for nausea, vomiting and diarrhoea.

## 4 DISCUSSION

This meta‐analysis of 38 randomized clinical trials, involving over 16 000 participants with T2D, demonstrated that newer incretin mimetics, particularly s.c. semaglutide and tirzepatide, provide greater reductions in HbA1c, FPG and body weight compared with earlier‐generation GLP‐1 RAs such as exenatide and lixisenatide. Despite their enhanced therapeutic efficacy, however, the incidence of GI AEs—specifically nausea, vomiting and diarrhoea—was not proportionally higher, suggesting that there is no strong relationship coupling larger desired therapeutic efficacy to significantly more GI AEs.

These findings build on previous evidence from head‐to‐head comparisons^7^, ^10^, ^11^, ^12^, ^13^, ^14^, ^15^, ^16^, ^17^, ^18^, ^19^ and network meta‐analyses^20^, ^21^ that demonstrated the superior efficacy of newer incretin mimetics. However, while prior studies noted an increased risk of GI AEs across all incretin‐based glucose‐lowering agents examined, the present analysis additionally demonstrates that the magnitude of therapeutic efficacy does not scale linearly with the effect size regarding therapeutic benefits. The overall ORs for nausea, vomiting and diarrhoea were 3.42, 4.07 and 1.83, respectively, with dose–response relationships observed within some agents but without substantial variability across compounds. Furthermore, regression analyses indicated that GI AEs accounted for less than 1.6% of the variability in efficacy outcomes, with highly efficacious agents such as semaglutide and tirzepatide exhibiting lower AE rates than expected based on their therapeutic efficacy.

One notable change to GLP‐1 RAs over time is the advancement in strategies to prolong the duration of action. Exenatide was the first to be introduced, administered twice daily.^24^ The other short‐acting compound, lixisenatide, was, nevertheless, introduced for once daily injections.^10^ Subsequent development of long‐acting GLP‐1 RAs aimed to provide stable pharmacokinetic profiles without troughs over a ≥ 24‐h period post administration.^2^, ^3^, ^25^ Followed by the once‐daily GLP‐1 RA, liraglutide (approved in 2014^26^), various strategies allowed a longer duration of action and stable pharmacokinetic profile. For example, weekly exenatide uses microsphere preparations for slow release,^12^ while semaglutide incorporates a fatty acid that binds to albumin, extending its half‐life.^27^, ^28^ Dulaglutide and albiglutide are modified GLP‐1 molecules attached to larger proteins—an immunoglobulin Fc fragment^29^ and albumin,^30^ respectively—further enhancing their duration of action. More recently, tirzepatide prolongs its activity through a free fatty diacid coupled to the bioactive peptide bearing sequence homology to both GIP and GLP‐1.^31^ Therefore, the observed differences in efficacy and tolerability can be attributed, in part, to advancements in pharmacokinetics and dosing regimens. Earlier GLP‐1 RAs, such as exenatide^24^ and lixisenatide,^10^ required more frequent dosing and had shorter half‐lives, which may have led to daily peak concentrations potentially contributing to a higher prevalence of AEs. In contrast, newer agents like semaglutide and tirzepatide have longer half‐lives and more gradual absorption profiles,^27^, ^32^ reducing peak plasma concentrations likely associated with GI AEs. Also, modern titration protocols, which involve lower initial doses and slower dose escalation, may enhance tolerability, allowing patients to reach higher maintenance doses with fewer AEs, although future studies should elucidate this interdependence further. In addition, preclinical studies^33^, ^34^ and a clinical trial^35^ have suggested that GIP agonism might mitigate nausea associated with GLP‐1 RA. However, this mechanism alone is unlikely to fully explain our observations, as a similar dissociation between therapeutic efficacy and GI adverse events was also seen with s.c. and oral semaglutide. More research is needed to determine the impact of co‐medications (e.g., SGLT‐2 inhibitors)^36^ and multiple mechanisms of actions (dual or triple agonists like tirzepatide) on the relationship between therapeutic efficacy and adverse events.

Of note, semaglutide is a unique compound available in both subcutaneous (s.c.) and oral formulations—the latter developed with an absorption enhancer to facilitate gastric uptake.^37^, ^38^ The therapeutic efficacy and GI intolerance associated with the currently approved highest dose of oral semaglutide (14 mg daily) appear to be lower than those observed with the highest approved dose of s.c. semaglutide (1.0 mg weekly). However, a Phase 2 trial demonstrated that higher doses of oral semaglutide (10, 20 and 40 mg) produced reductions in HbA1c, FPG and body weight comparable with those seen with 1.0 mg weekly s.c. semaglutide.^38^ These findings suggest that, should future development support the approval of higher‐dose oral semaglutide, it may offer an effective alternative for patients who prefer non‐injectable therapies for diabetes management. However, additional studies would be needed to evaluate the relationship between therapeutic efficacy and GI intolerance with higher doses of oral semaglutide compared with its subcutaneous formulation.

While GI side effects are the most common AEs associated with GLP‐1 RAs, strategies to mitigate these effects have been recommended. For example, the American Diabetes Association (ADA) advises that GLP‐1 RAs should be titrated slowly to mitigate these GI side effects, particularly in older adults or those with pre‐existing GI conditions.^39^ Similarly, the American College of Cardiology (ACC) recommends gradual dose escalation and patient education on meal size to reduce the incidence of these adverse effects.^40^ Given the substantial weight loss, glycaemic and cardiovascular benefits consistently demonstrated in randomized, placebo‐controlled clinical trials (RCTs) of long‐acting GLP‐1 RAs, accurately understanding the trade‐offs is essential for practicing evidence‐based medicine in this high‐risk population. In this regard, the current study supports the use of newer incretin mimetics as first‐line options for patients with T2D, particularly those with obesity or difficulty achieving glycaemic control with other therapies. Given their greater efficacy and comparable tolerability, semaglutide and tirzepatide offer a favourable benefit–risk profile that can help address the dual challenges of hyperglycaemia and excess weight. Moreover, the absence of a strong correlation between efficacy and AE rates suggests that clinicians can prioritize more efficacious agents without substantially increasing the risk of treatment discontinuation due to GI AEs.

The strengths of this study include its comprehensive analysis of phase 3 and 4 RCTs, robust sample size and rigorous methodological approach using pooled weighted means and ORs to assess both efficacy and safety outcomes. The inclusion of various doses and formulations allowed for a nuanced examination of dose–response relationships and their impact on GI AEs.

However, some limitations should be noted. The analysis relied on aggregate data rather than individual patient data, which limits the ability to explore patient‐specific factors such as sex and race/ethnicity that may influence variability in therapeutic response and adverse events. Second, heterogeneity in study designs, background therapies other than insulin and trial durations may have influenced the observed outcomes. Third, we reported GI tolerability using ORs for proportions of study participants reporting AEs of interest rather than absolute event rates. This decision was made because absolute event rates are inconsistently reported across trials and comparing unannualized event rates would be prone to bias due to differences in trial duration and adverse event ascertainment. We acknowledge, however, that OR calculations may be influenced by trials with zero prevalence of AEs in placebo arms.^41^, ^42^, ^43^ That said, such effects are likely partially mitigated by other trial arms of the same compound and dose. Fourth, the analysis is based exclusively on RCTs. While this confers methodological rigour, the generalizability of our findings to real‐world settings—and the accurate estimation of safety signals in routine clinical care—will require further, long‐term studies. Lastly, while GI AEs were the primary focus, other non‐GI safety outcomes, such as hypoglycaemia, were not assessed in the current analysis, limiting the study's scope regarding the overall safety profile of incretin mimetics.

In conclusion, this meta‐analysis highlights the superior efficacy and relatively favourable tolerability profile of newer incretin mimetics, reinforcing their role as key therapeutic options for managing T2D. While GI AEs should be carefully monitored when initiating and titrating incretin mimetics, nevertheless, the reassuring dissociation between therapeutic efficacy and GI AEs observed in this study underscores the importance of individualized treatment selection and titration strategies to maximize patient outcomes.

## AUTHOR CONTRIBUTIONS

YMK, VP and MAN designed the study. YMK, VP, SL and MAN analysed the data, performed the statistical analysis and wrote the manuscript. All authors have seen and approved the final draft of this manuscript and have decided to submit it for publication. MAN is the guarantor who takes full responsibility for the work, including study design, access to data and the decision to submit and publish the manuscript.

## FUNDING INFORMATION

YMK is funded by a T32 postdoctoral training grant from the National Institute of Diabetes and Digestive and Kidney Diseases (5T32DK007529).

## CONFLICT OF INTEREST STATEMENT

YMK and VP have nothing to disclose. SL is an advisory board member for Novo Nordisk and AstraZeneca and has served on the speakers' bureau of Novo Nordisk, Sanofi, Boehringer Ingelheim, AstraZeneca and MSD. He has received research funding from CKD and Daewoong Pharma. MAN has been member on advisory boards or has consulted with Boehringer Ingelheim, Eli Lilly & Co., Medtronic, Merck, Sharp & Dohme, Novo Nordisk, Pfizer, Regor, Sun Pharma and Structure Therapeutics (ShouTi, Gasherbrum). He has received grant support from Merck, Sharp & Dohme. He has also served on the speakers' bureau of Eli Lilly & Co., Menarini/Berlin Chemie, Merck, Sharp & Dohme, Medscape, Medical Learning Institute and Novo Nordisk. SL is an advisory board member for Novo Nordisk and AstraZeneca and has served on the speakers' bureau of Novo Nordisk, Sanofi, Boehringer Ingelheim, AstraZeneca and MSD. He has received research funding from Chong Kun Dang and Daewoong Pharma.

## PEER REVIEW

The peer review history for this article is available at https://www.webofscience.com/api/gateway/wos/peer-review/10.1111/dom.16398.

## Supporting information


**Data S1.** Supporting information.

## Data Availability

Data extracted from included studies; data used for all analyses and any other materials used in the review can be accessed upon reasonable request to the corresponding author and at the discretion of the authors.
