# Supplementary material for: Comparative efficacy and tolerability of currently approved incretin mimetics: A systematic analysis of placebo‐controlled clinical trials
Source: Diabetes Obes Metab. 2025 Apr 11;27(7):3736–46. doi: 10.1111/dom.16398 (PMC12146062; doi:10.1111/dom.16398)
Supplement: Supplementary file 1 — Data S1. Supporting information. [file DOM-27-3736-s001.docx]

**Supplementary Appendix**

**Comparative Effectiveness and Tolerability of Currently Approved Incretin Mimetics:
A Systematic Analysis of Placebo-Controlled Clinical Trials**

Yu Mi Kang, Viktoria Punov, Soo Lim, and Michael A. Nauck

| **Table of Contents** | **Page** |
| --- | --- |
| **Supplementary Table 1.** Clinical trials studying clinical effects of GLP-1 receptor agonists and the dual GIP/GLP-1 co-agonist tirzepatide compared to placebo treatment in subjects with type 2 diabetes and providing data for the present systematic analysis | 2 |
| **Supplementary Figure 1.** Preferred Reporting Items for Systemic Reviewers and Meta-analyses (PRISMA) Flow Diagram | 3 |
| **Supplementary Figure 2.** Review of included studies using Cochrane Risk-of-Bias Assessment Tool Version 2 | 4 |
| **Supplementary Table 2.** Heterogeneity in the pooled effect sizes of therapeutic efficacy outcomes was low across study arms involving the highest approved doses of each incretin mimetic | 5 |
| **Supplementary Figure 3.** Comparative effectiveness in controlling glycemia and promoting body weight reduction of approved incretin mimetics in studies using (basal) insulin as a comedication or not | 6 |
| **Supplementary Figure 4.** Dose-response relationships regarding the achievement of HbA1c targets < 7.0 % or ≤ 6.5 % with approved incretin mimetics | 7 |
| **Supplementary Figure 5.** Dose-response relationships regarding the achievement of HbA1c targets < 7.0 % or ≤ 6.5 % with approved incretin mimetics. | 8 |
| **Supplementary Figure 6.** Diarrhea reported in clinical studies of approved incretin mimetics | 9 |
| **Supplementary Figure 7.** Discontinuation of randomized treatment (for any reason or because of adverse events) reported in clinical studies of approved incretin mimetic | 10 |
| **Supplementary Figure 8.** Relationship between an increased risk for reporting diarrhea and efficacy regarding placebo-subtracted reductions in HbA_1c_, fasting plasma glucose, and body weight | 11 |
| **Supplementary Figure 9.** Relationship between an increased risk for discontinuing the randomized treatment (for any reason or because of adverse events) and efficacy regarding placebo-subtracted reductions in HbA_1c_, fasting plasma glucose, and body weight | 12 |

**Supplementary Table 1.** Clinical trials studying clinical effects of GLP-1 receptor agonists and the dual GIP/GLP-1 co-agonist tirzepatide compared to placebo treatment in subjects with type 2 diabetes and providing data for the present systematic analysis

| Study number | Compound | Publication | Dosage | Study Acronym | Duration (weeks) |  |
| --- | --- | --- | --- | --- | --- | --- |
| 1 | Exenatide b.i.d. | Buse et al. 2004 | 5 or 10 µg twice daily | AMIGO 1 | 30 |  |
| 2 |  | DeFronzo et al. 2005 | 5 or 10 µg twice daily | AMIGO 2 | 30 |  |
| 3 |  | Kendall et al. 2005 | 5 or 10 µg twice daily | AMIGO 3 | 30 |  |
| 4 | Lixisenatide | G. B. Bolli et al. 2013 | 20 µg per once daily | GetGoal 1 | 24 |  |
| 5 |  | Fonseca et al. 2012 | 20 µg once daily | GetGoal Mono | 12 |  |
| 6 |  | Ahren et al. 2013 | 20 µg once daily | GetGoal-M | 24 |  |
| 7 |  | Pinget et al. 2013 | 20 µg once daily | GetGoal-P | 24 |  |
| 8 |  | Seino et al. 2012 | 20 µg once daily | GetGoal -L-Asia | 24 |  |
| 9 |  | Riddle et al. 2013 | 20 µg once daily | GetGoal-L | 24 |  |
| 10 |  | Bolli et al. 2014 | 20 µg once daily | GetGoal-F 1 | 24 |  |
| 11 |  | Pan et al. 2014 | 20 µg once daily | GetGoal-M-Asia | 24 |  |
| 12 |  | Rosenstock et al. 2014 | 20 µg once daily | GetGoal-S | 24 |  |
| 13 | Liraglutide | Marre et al. 2009 | 0.6, 1.2, or 1.8 mg per day | LEAD 1 | 26 |  |
| 14 |  | Nauck et al. 2009 | 0.6, 1.2, or 1.8 mg per day. | LEAD 2 | 26 |  |
| 15 |  | Bernard Zinman | 1.2, or 1.8 mg per day | LEAD 4 | 26 |  |
| 16 |  | Russell-Jones et al. 2009 | 1.8 mg per day | LEAD 5 | 26 |  |
| 17 |  | Blonde et al. 2020 | 1.8 mg per day | LIRA ADD2SGLT2 | 26 |  |
| 18 | Exenatide q.w. | Gadde et al. 2017 | 2 mg per week | DURATION NEO 2 | 28 |  |
| 19 |  | Guja et al. 2018 | 2 mg per week | DURATION 7 | 28 |  |
| 20 | Dulaglutide | Wysham et al. 2014 | 0.75 or 1.5 mg per week | AWARD 1 | 26 |  |
| 21 |  | Skrivanek et al. 2014 | 0.75 or 1.5 mg per week | AWARD 5 | 26 |  |
| 22 |  | Dungan et al. 2016 | 1.5 mg per week | AWARD 8 | 24 |  |
| 23 |  | Pozzilli et al. 2017 | 1.5 mg/ per week | AWARD 9 | 28 |  |
| 24 |  | Ludvik et al. 2018 | 0.75 or 1.5 mg per week | AWARD 10 | 24 |  |
| 25 | Albiglutide | Reusch et al. 2014 | 30 mg per week | HARMONY 1 | 52 |  |
| 26 |  | Nauck et al. 2016 | 30 or 50 mg per week | HARMONY 2 | 52 |  |
| 27 |  | Åhren et al. 2014 | 30 mg per week | HARMONY 3 | 104 |  |
| 28 |  | Home et al. 2015 | 30 mg per week | HARMONY 5 | 52 |  |
| 29 | Semaglutide s.c. | Sorli et al. 2017 | 0.5 or 1 mg per week | SUSTAIN 1 | 30 |  |
| 30 |  | Rodbard et al. 2018 | 0.5 or 1 mg per week | SUSTAIN 5 | 30 |  |
| 31 |  | Zinman et al. 2019 | 0.5 mg per week | SUSTAIN 9 | 30 |  |
| 32 | Semaglutide oral | Aroda et al. 2019 | 3, 7, or 14 mg per day | PIONEER 1 | 26 |  |
| 33 |  | Pratley et al. 2019 | 14 mg per day | PIONEER 4 | 52 |  |
| 34 |  | Mosenzon et al. 2019 | 14 mg per day | PIONEER 5 | 26 |  |
| 35 |  | Zinman et al. 2019 | 3, 7, or 14 mg per day | PIONEER 8 | 26 |  |
| 36 |  | Yamada et al. 2020 | 3, 7, or 14 mg per day | PIONEER 9 | 52 |  |
| 37 | Tirzepatide | Rosenstock et al. 2021 | 5, 10, or 15 mg per week | SURPASS-1 | 40 |  |
| 38 |  | Dahl et al. 2022 | 5, 10, or 15 mg per week | SURPASS-5 | 40 |  |
| Studies were selected form the pivotal clinical trial program supporting approval of the respective GLP-1 receptor agonist or dual GIP/GLP-1 receptor co-agonist tirzepatide (identified by the typical acronym for each compound). Only studies providing placebo-subtracted effect sizes for the main outcome parameters HbA_1c_, fasting plasma glucose concentration, and body weight reduction were selected. | | | | | | |

**
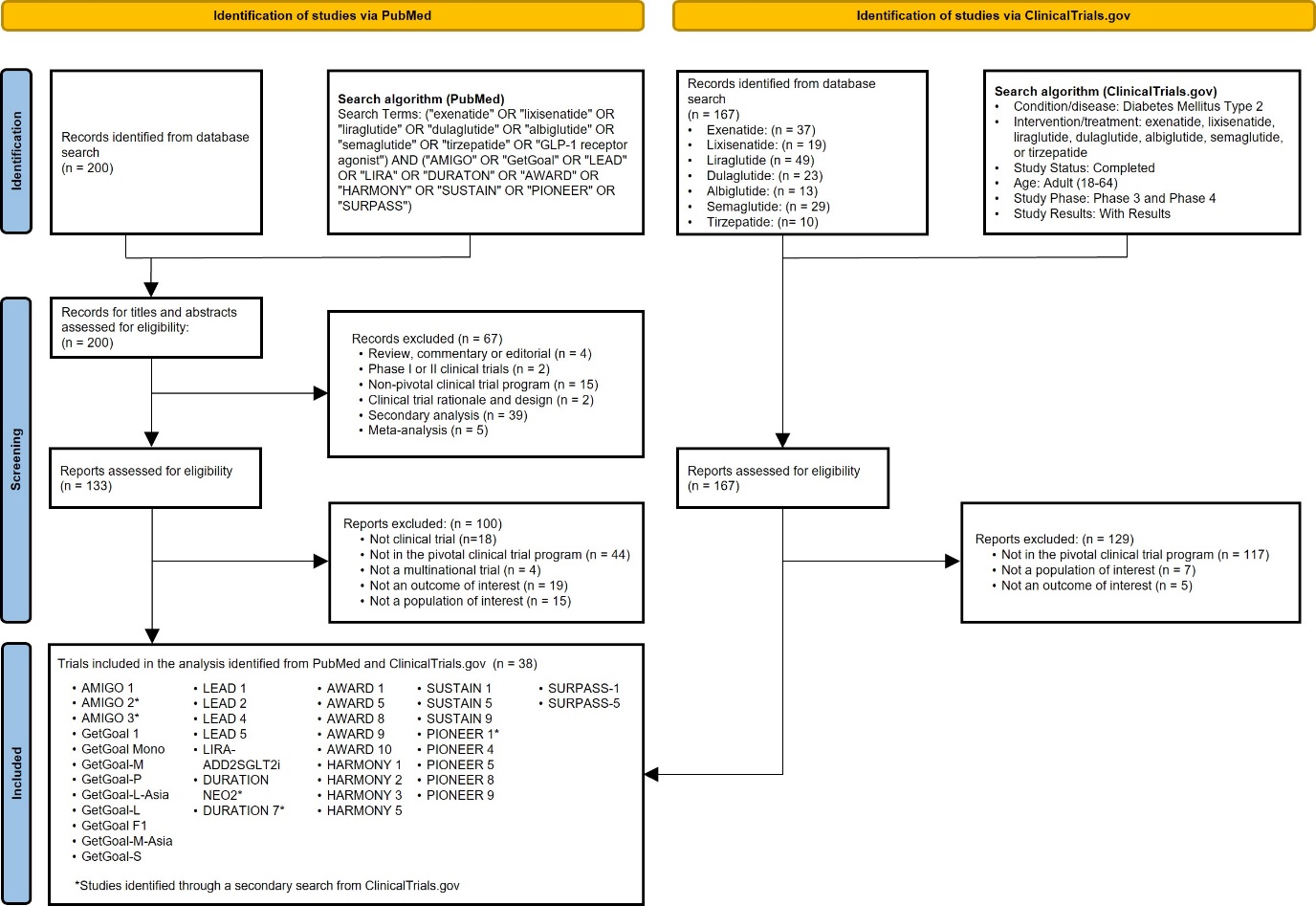
**

**Supplementary Figure 1. Preferred Reporting Items for Systemic Reviewers and Meta-analyses (PRISMA) Flow Diagram.** PRISMA Flow Diagram of the search process, selection, and exclusion of publications. Studies were initially identified through a systematic PubMed search resulting in 32 pivotal programs using a query involving the compound or program name (left). This search was supplemented by an independent search using ClinicalTrials.gov (right), which identified five additional studies (AMIGO2, AMIGO3, DURATION-7, DURATION-NEO2, and PIONEER1 with an asterisk [*]). A total of 38 studies met our search criteria as of March 9, 2024, and were included in this meta-analysis.


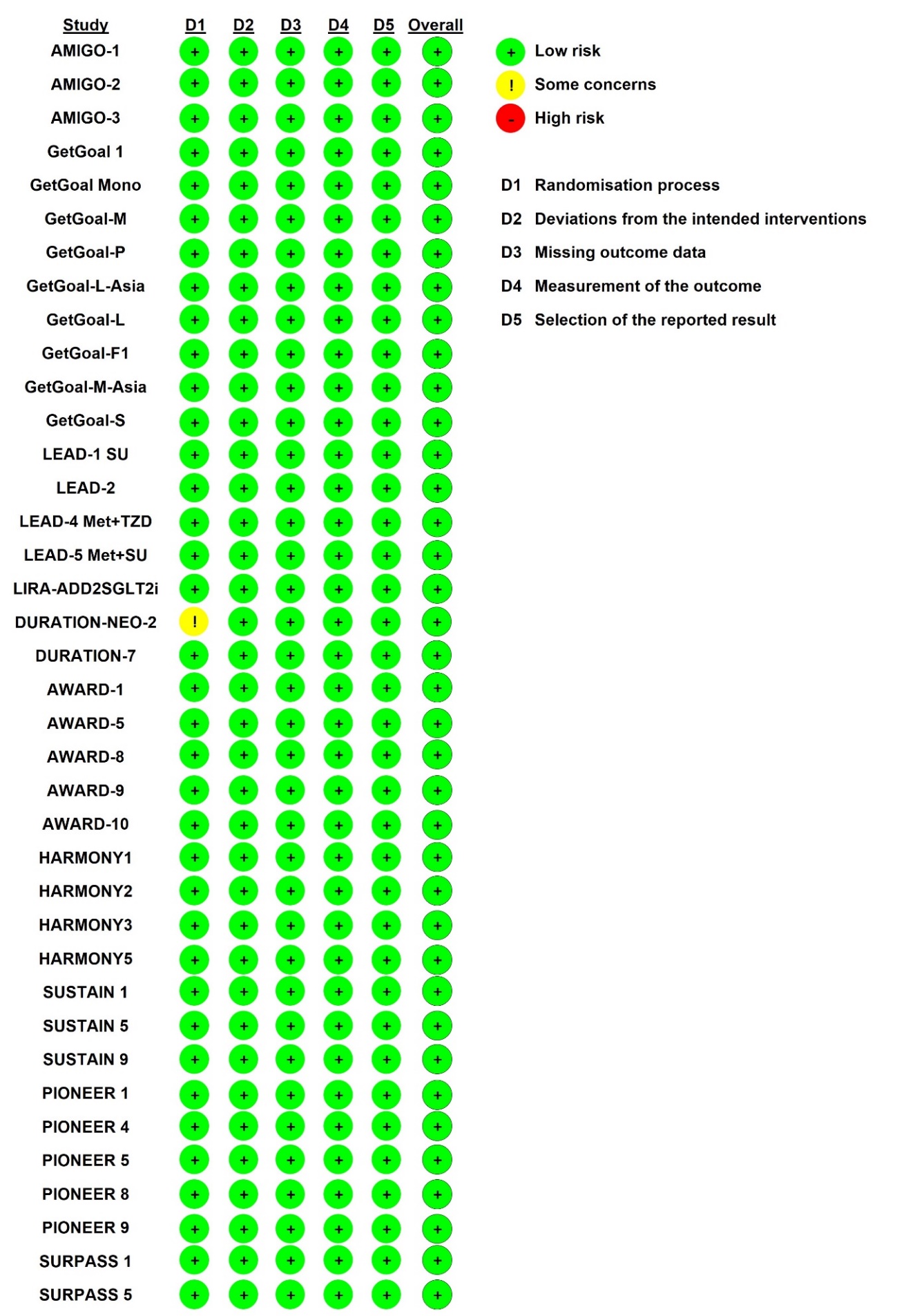
**Supplementary Figure 2. Review of included studies using Cochrane Risk-of-Bias Assessment Tool Version 2**. Assessment of risk of bias using the Risk of Bias 2.0 tool from the Cochrane Collaboration for studies included in the present meta-analysis.

**Supplementary Table 2. Heterogeneity in the pooled effect sizes of therapeutic efficacy outcomes was low across study arms involving the highest approved doses of each incretin mimetic.** Heterogeneity for pooled effect sizes of efficacy measures (placebo-subtracted reductions in HbA_1c_, fasting plasma glucose, and body weight vs. baseline) for the highest doses of GLP-1 receptor agonists or the dual GIP/GLP-1 receptor agonist tirzepatide was assessed using random-effects meta-analyses model.

|  |  | Placebo-Subtracted Changes  in HbA1c | | | | |  | Placebo-Subtracted Changes  in Fasting Plasma Glucose | | | | |  | Placebo-Subtracted Changes  in Body Weight | | | | |
| --- | --- | --- | --- | --- | --- | --- | --- | --- | --- | --- | --- | --- | --- | --- | --- | --- | --- | --- |
| Compound/preparation |  | **Tau^2^** | **Q** | **df** | **P for df** | **I^2^ (%)** |  | **Tau^2^** | **Q** | **df** | **P for df** | **I^2^ (%)** |  | **Tau^2^** | **Q** | **df** | **P for df** | **I^2^ (%)** |
| Exenatide 10 µg b.i.d. |  | 0 | 1.68 | 2 | 0.4316 | 0.00 |  | 0.4872 | 7.67 | 2 | 0.0216 | 73.90 |  | 0 | 0.73 | 2 | 0.6959 | 0.00 |
| Lixisenatide 20 µg/day |  | 0.0638 | 66.12 | 10 | <0.0001 | 84.90 |  | 0.0669 | 15.54 | 8 | 0.0494 | 48.50 |  | 0.0118 | 12.2 | 10 | 0.2716 | 18.10 |
| Liraglutide 1.8 mg/day |  | 0.0806 | 29.67 | 4 | <0.0001 | 86.50 |  | 2.282 | 63.08 | 4 | <0.0001 | 93.70 |  | 0.3552 | 27.19 | 4 | <0.0001 | 85.30 |
| Exenatide 2 mg/week |  | 0 | 0 | 1 | 0.9667 | 0.00 |  | 1.1391 | 9.09 | 1 | 0.0026 | 89.00 |  | 0 | 0.01 | 1 | 0.931 | 0.00 |
| Dulaglutide 1.5 mg/week |  | 0.0308 | 11.73 | 4 | 0.0194 | 65.90 |  | 0.699 | 23.15 | 4 | 0.0001 | 82.70 |  | 0.1886 | 14.08 | 3 | 0.0028 | 78.70 |
| Albiglutide 50 mg/week* |  | NA | NA | NA | NA | NA |  | NA | NA | NA | NA | NA |  | NA | NA | NA | NA | NA |
| Semaglutide s.c. 1 mg/week |  | 0.0162 | 4.22 | 2 | 0.1215 | 52.60 |  | 0.3509 | 4.58 | 2 | 0.101 | 56.40 |  | 0 | 1.64 | 2 | 0.4407 | 0.00 |
| Oral semaglutide 14 mg/day |  | 0.0311 | 15.98 | 4 | 0.003 | 75.00 |  | 0.7815 | 20.76 | 4 | 0.0004 | 80.70 |  | 0 | 2.34 | 3 | 0.505 | 0.00 |
| Tirzepatide 15 mg/week |  | 0.1871 | 11.57 | 1 | 0.0007 | 91.40 |  | 0.778 | 2.17 | 1 | 0.1411 | 53.80 |  | 2.2429 | 33.82 | 1 | <0.0001 | 97.00 |

*There was only one highest dose arm for albiglutide (50mg weekly) in the included studies, limiting heterogeneity assessment.

Abbreviations: df, degrees of freedom; NA, not available; Q, Cochran’s Q (Chi^2^),

**Supplementary Figure 3. Comparative effectiveness in controlling glycemia and promoting body weight reduction of approved incretin mimetics in studies using (basal) insulin as a comedication or not.** Pooled effect sizes are displayed for placebo-controlled studies with the highest approved doses of exenatide b.i.d., lixisenatide, liraglutide, exenatide q.w., dulaglutide, albiglutide, semaglutide s.c., semaglutide p.o., and tirzepatide. Placebo-subtracted (A) changes in HbA_1c_ and (B) body weight; (C) fasting plasma glucose concentrations and (D) proportions achieving an HbA_1c_ < 7.0 % (< 53 mmol/mol) (all vs. baseline values) are shown as x-fold values ± 95 % confidence intervals compared to results obtained for lixisenatide 20 µg per day (the reference treatment). Results from studies not allowing (basal) insulin as a comedication are shown by columns with grey frames and 95 % CI bars, those from studies allowing (basal) insulin, by columns with black frames and 95 % CI bars (see legend). Statistical analysis: Non-overlapping 95 % confidence intervals indicate significant differences. N.r.: Not reported (no such studies were available)

**Supplementary Figure 4. Dose-response relationships regarding the effectiveness in controlling glycemia and promoting body weight reduction of approved incretin mimetics.** Pooled effect sizes are displayed for placebo-controlled studies with all approved doses of exenatide b.i.d., lixisenatide, liraglutide, exenatide q.w., dulaglutide, albiglutide, semaglutide s.c., semaglutide p.o., and tirzepatide. Placebo-subtracted (A) reductions in HbA_1c_ and (B) fasting plasma glucose concentrations and (D) body weight reductions (all vs. baseline values) are shown. Proportion of participants achieving an HbA_1c_ < 7.0 % (<53 mmol/mol) is shown for actively treated patients (see colour code) as well as for those receiving placebo treatment (in grey; C). Mean values ± standard errors of the mean. Statistical analysis: Student’s t-test for continuous variables, comparing either results between the lowest and highest available doses of a given compound/preparation. Asterisks (*) indicate a significant difference (p < 0.05) to placebo treatment; daggers (†) indicate a significant difference (p < 0.05) between the highest and lowest dose of the respective agent/preparation indicating a significant dose-response relationship.


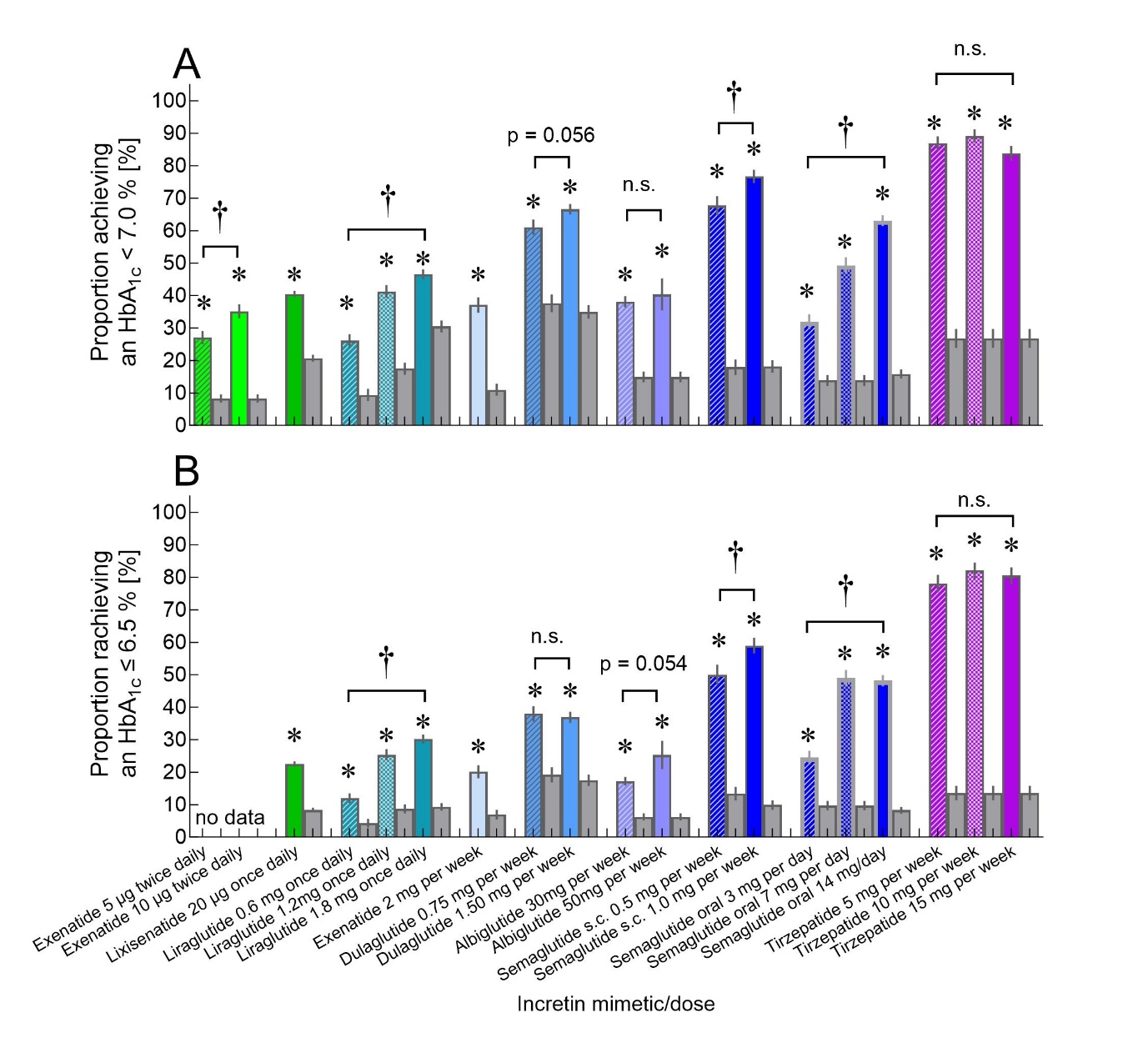


**Supplementary Figure 5. Dose-response relationships regarding the achievement of HbA1c targets < 7.0 % or ≤ 6.5 % with approved incretin mimetics.** Pooled effect sizes are displayed for placebo-controlled studies with all approved doses of exenatide b.i.d., lixisenatide, liraglutide, exenatide q.w., dulaglutide, albiglutide, semaglutide s.c., semaglutide p.o., and tirzepatide. Proportions of patients achieving an HbA_1c_ < 7.0 % (53 mol/mol; B) and ≤ 6.5 % (48 mmol/mol; D) are shown for actively treated patients (see colour code) as well as for those receiving placebo treatment (in grey). Mean values ± standard errors of the mean. Statistical analysis: Fisher’s exact test for 2 x 2 tables, comparing either active treatment with placebo treatment (for each agent/preparation), or results between the lowest and highest available doses of a given compound/preparation or. Asterisks (*) indicate a significant difference (p < 0.05) to placebo treatment; daggers (†) indicate a significant difference (p < 0.05) between the highest and lowest dose of the respective agent/preparation indicating a significant dose-response relationship.

**Supplementary Figure 6. Diarrhea reported in clinical studies of approved incretin mimetics.** Pooled effect sizes are displayed for placebo-controlled studies with all approved doses of exenatide b.i.d., lixisenatide, liraglutide, exenatide q.w., dulaglutide, albiglutide, semaglutide s.c., semaglutide p.o., and tirzepatide. Proportions reporting diarrhea (A) are shown for actively treated patients (see colour code) as well as for those receiving placebo treatment (in grey). Mean values ± standard errors of the mean. In addition, odds ratios (and their 95 % confidence intervals) are shown for active vs. placebo treatment (B). Statistical analysis: Contingency table analysis (Fisher’s exact test for 2 x 2 tables) comparing either results active treatment with placebo treatment or between the lowest and highest available doses of a given compound/preparation. Asterisks (*) indicate a significant difference (p < 0.05) to placebo treatment; daggers (†) indicate a significant difference (p < 0.05) between the highest and lowest dose of the respective agent/preparation indicating a significant dose-response relationship.

**Supplementary Figure 7. Discontinuation of randomized treatment (for any reason or because of adverse events) reported in clinical studies of approved incretin mimetics.** Pooled effect sizes are displayed for placebo-controlled studies with all approved doses of exenatide b.i.d., lixisenatide, liraglutide, exenatide q.w., dulaglutide, albiglutide, semaglutide s.c., semaglutide p.o., and tirzepatide. Proportions reporting discontinuation of randomized treatment for any reason (A) or because of adverse events (C) are shown for actively treated patients (see colour code) as well as for those receiving placebo treatment (in grey). Mean values ± standard errors of the mean. In addition, odds ratios (and their 95 % confidence intervals) are shown for active vs. placebo treatment for discontinuation of randomized treatment for any reason (B) and because of adverse events (D). Statistical analysis: Contingency table analysis (Fisher’s exact test for 2 x 2 tables) comparing either results active treatment with placebo treatment or between the lowest and highest available doses of a given compound/preparation. Asterisks (*) indicate a significant difference (p < 0.05) to placebo treatment; daggers (†) indicate a significant difference (p < 0.05) between the highest and lowest dose of the respective agent/preparation indicating a significant dose-response relationship.

**Supplementary Figure 8. Relationship between an increased risk for reporting diarrhea and efficacy regarding placebo-subtracted reductions in HbA_1c_, fasting plasma glucose, and body weight.** On the x-axis, the odds ratios (± standard errors of the means calculated from 95 % confidence intervals) for reporting diarrhea with active treatment relative to placebo treatment is shown for each agent/preparation/dose of incretin mimetics. On the y-axis, pooled effect sizes are displayed for placebo-subtracted reductions in HbA_1c_ (A), fasting plasma glucose (B), and body weight (B) reported in studies with all approved doses of exenatide b.i.d., lixisenatide, liraglutide, exenatide q.w., dulaglutide, albiglutide, semaglutide s.c., semaglutide p.o., and tirzepatide. The results of linear regression analysis are displayed as the regression line (± 95 % confidence intervals, in red), the regression equation, the coefficient of correlation squared (r^2^), and the related p-value.

**Supplementary Figure 9. Relationship between an increased risk for discontinuing the randomized treatment (for any reason or because of adverse events) and efficacy regarding placebo-subtracted reductions in HbA_1c_, fasting plasma glucose, and body weight.** On the x-axis, the odds ratios (± standard errors of the means calculated from 95 % confidence intervals) for discontinuing randomized treatment for any reason (A-C) or because of adverse events (D-F) with active treatment relative to placebo treatment is shown for each agent/preparation/dose of incretin mimetics. On the y-axis, pooled effect sizes are displayed for placebo-subtracted reductions in HbA_1c_, fasting plasma glucose, and body weight reported in studies with all approved doses of exenatide b.i.d., lixisenatide, liraglutide, exenatide q.w., dulaglutide, albiglutide, semaglutide s.c., semaglutide p.o., and tirzepatide. The results of linear regression analysis are displayed as the regression line (± 95 % confidence intervals, in red), the regression equation, the coefficient of correlation squared (r^2^), and the related p-value.
